# Electroencephalogram activity related to psychopathological and neuropsychological symptoms in institutionalised minors: a systematic review

**DOI:** 10.1017/neu.2025.19

**Published:** 2025-05-08

**Authors:** Carlos Barbosa-Torres, Natalia Bueso-Izquierdo, Alejandro Arévalo-Martínez, Juan Manuel Moreno-Manso

**Affiliations:** Department of Psychology & Anthropology, University of Extremadura, Badajoz, Spain

**Keywords:** Electroencephalogram, minors, psychopathology, neuropsychology, psychosocial deprivation

## Abstract

**Objective:**

This systematic review aims to update the current evidence on the effects of institutionalisation in minors living in residential care homes, specifically focusing on alterations in neuronal systems and their association with psychopathological and neuropsychological outcomes.

**Methods:**

Searches were conducted in the Web of Science, Scopus, PubMed, and Google Scholar databases, following PRISMA methodology for peer-reviewed empirical articles. The final selection comprised 10 studies that met the inclusion criteria: (1) published articles with quantitative data, (2) aimed at observing the relationship between psychological and neuropsychological symptoms and the electroencephalogram (EEG) activity in institutionalised children, (3) published between 2016 and 2023, and (4) examining institutionalised minors in residential care homes.

**Results:**

The articles show that these children exhibit general immaturity in EEG patterns, with a predominance of slow waves (primarily in the theta band). They also demonstrate poorer performance in executive functions (e.g. working memory, inhibition, and processing speed) and cognitive processes, along with a higher risk of externalising problems. However, current evidence does not allow definitive conclusions on whether early EEG abnormalities predict long-term neuropsychological deficits, despite data showing associations between EEG changes and certain cognitive dysfunctions at the time of evaluation.

**Conclusion:**

The reviewed evidence suggests that EEG alterations in institutionalised minors are linked to executive dysfunction and increased psychopathological risk. These findings highlight the value of EEG in identifying at-risk children and inform the design of preventive interventions. Longitudinal studies are needed to clarify causal relationships.

Significant outcomes

- Institutionalised minors show electroencephalogram immaturity, particularly increased slow-wave activity (e.g. theta), which has been linked to cognitive and executive dysfunctions.
- Early psychosocial deprivation impacts the development of brain regions associated with executive functions, increasing the risk of externalising psychopathological symptoms.
- Reducing the duration of institutionalisation and improving foster care quality are key factors in mitigating long-term cognitive and neuropsychological consequences.

Limitations

- The limited number of studies meeting the inclusion criteria restricts the generalisability of findings across different institutional settings.
- The predominance of cross-sectional designs hinders conclusions regarding the long-term effects of electroencephalogram (EEG) immaturity on cognitive development.
- The exclusion of doctoral and master’s theses, as well as studies using alternative EEG methodologies, may have led to the omission of valuable insights on the topic.

## Introduction

The emphasis for neglected minors in residential care homes has traditionally been on basic care, often characterised by a high child-to-educator ratio, insufficient social and cognitive stimulation, and limited emotional support (Mason and Narad, 2005; Nweze *et al*., 2022). Currently, between 2.5 and 8 million minors find themselves in institutions worldwide, while 1.3 million live with foster families (Petrowski *et al*., 2017; Hillis *et al*., 2021; UNICEF, 2024). The absence of educational and therapeutic support, or inadequate support during institutionalisation, can have lasting, damaging effects on children’s neurocognitive development, especially when it occurs at a young age (Fox *et al*., 2010). Children in residential care run a greater risk of suffering negative consequences in their emotional and psychological development compared to minors who are not institutionalised. The place and conditions of social coexistence play an important role in the minors’ maturation. Different studies indicate that the cognitive processes of minors in residential care are significantly lower than those of children who live with their families in a normative environment (Johnson *et al*., 2010; Lloyd and Barth, 2011). Similarly, the minors’ development can deteriorate even further when such circumstances as abuse also occur at the same time (Herringa *et al*., 2013), as happens with a high percentage of institutionalised children, who suffered abuse in their families or in their care homes (Bick and Nelson, 2016).

Various research studies point out that psychosocial deprivation in the context of residential care is related to disturbances in several cognitive processes, such as a reduced IQ in institutionalised minors (Van Ijzendoorn *et al*., 2008) or the lack of linguistic competence and accuracy in the use of semantics (Kumar *et al*., 2014; Chinn *et al*., 2023). However, it is not only in the domains of intelligence and language where disorders can occur but also through a high prevalence towards psychopathological symptoms and disorders (Gunnar and Reid, 2019). The most common problems are symptoms of internalising disorders, such as anxiety and depression, externalising disorders, and other problems related to the processing and expression of emotions (Humphreys *et al*., 2015; Young *et al*., 2017), which tend to increase with age (Sonuga-Barke *et al*., 2017). Nevertheless, some of the most consistent findings are the effects of institutional deprivation on the deterioration of attention abilities and executive functions in general (Pollak *et al*., 2010; McDermott *et al*., 2013). It has also been connected to specific deficits in the executive functions, such as inhibitory control (McDermott *et al*., 2012), response or error monitoring (Loman *et al*., 2013), and problems of attention (Espano, 2013).

Over the last few decades, neuroimaging studies have been applied to the analysis of the general and specific neurological implications of the impact of institutional psychosocial deprivation (Sheridan *et al*., 2012; Olson *et al*., 2015). At a structural level, it has been demonstrated that institutionalised children present lower total brain, grey, and white matter volumes compared to non-institutionalised minors, in addition to having a smaller head circumference (Hodel *et al*., 2015; Sheridan *et al*., 2022). Nevertheless, it is on a functional level that we find particularly relevant data for comparing basic and higher-order psychological processes (VanTieghem *et al*., 2021). One of the techniques traditionally used to reflect the intrinsic dynamics of brain activity in repose is the electroencephalogram (EEG) (Anderson and Perone, 2018). This technique has been used as a biomarker of brain functioning in both cross-sectional studies (Vanderwert *et al*., 2010; Nagamitsu *et al*., 2011; Debnath *et al*., 2020) and large-scale longitudinal studies (Norton *et al*., 2021; Troller-Renfree *et al*., 2022).

In typically developing children, resting-state brain activity shows a decline in the power of low-frequency bands, such as theta, alongside a progressive increase in higher-frequency bands, including alpha, beta, and gamma, from infancy through the age of eleven (Uhlhaas *et al*., 2010). This activity profile is also associated with specific skills. For instance, higher absolute power in the medium and high-frequency bands, such as alpha, beta, and gamma, has been associated with stronger language skills (Maguire and Schneider, 2019), high cognitive skills (Williams *et al*., 2012), and good socio-emotional relationships (Brito *et al*., 2019). The individual electroencephalographic differences found in institutionalised minors have been associated with distinct cognitive and behavioural outcomes. For example, the presence of greater absolute or relative low-frequency power (e.g. theta) has been linked to the development of behavioural, attention, and learning problems (Barry *et al*., 2003; McLaughlin *et al*., 2011).

Although there have been many studies concerning the effects for minors suffering from neglect on a behavioural level, the effects on the underlying neuronal systems from behavioural and emotional deficits, as well as institutional psychosocial deprivation, have rarely been investigated. It is still unclear whether individual differences in resting brain activity are significantly related to long-term variations in cognitive processes. Thus, this review studies the EEG activity in institutionalised minors and its relation to psychopathological and neuropsychological symptoms, analysing the possible long-term consequences.

## Method

This systematic review adhered to the PRISMA guidelines (Preferred Reporting Items for Systematic Reviews and Meta-Analyses) (Page *et al*., 2021) and is detailed in Supplementary Materials, Table S1.

### Selection criteria for articles

We used the following criteria to determine whether studies were eligible for inclusion: (1) scientific articles with quantitative data; (2) studies primarily aimed at observing the relationship between psychological and neuropsychological symptoms and the EEG spectrum in institutionalised children; (3) studies published in English between 2016 and 2023, as the last updated review on this topic was published at the end of 2015 (Perego *et al*., 2016); and (4) only studies in which the population consisted of children institutionalised in centres for minors or in foster care were considered, regardless of the presence of a control/comparison group. Studies where the population resided in residential health centres, intensive care units, operating rooms, or hospitals were excluded. Furthermore, studies were excluded if the sample consisted of children or adolescents with specific disorders or pathologies (e.g. intellectual disabilities and other neurodevelopmental disorders, cognitive impairments, autism, oncological conditions, traumatic brain injuries, sleep disorders, epilepsy, seizure disorders, and other neurological disorders, or alcohol abuse). Additionally, we excluded articles if the EEG measurements assessed cognitive processes outside the scope of this study. We also excluded reviews, doctoral theses, and undergraduate or master’s theses, focusing exclusively on peer-reviewed journal articles to ensure high methodological rigor and reliability. Finally, studies were excluded if the title, abstract, or keywords did not clearly indicate relevance to the research objective.

### Search strategy and quality assessment

The search for articles to include in the review was carried out in the Web of Science, Scopus, PubMed, and Google Scholar databases in February 2024. The eligibility criteria were kept broad to encompass the range of empirical studies that met the inclusion criteria. The algorithm used for papers in each database was as follows: for example, Search ‘institutionalized care’ [OR] ‘residential care’ [OR] ‘foster care’ [AND] ‘child’ [OR] ‘child institutionalized’ [AND] ‘electroencephalogram’ [OR] ‘EEG’ [OR] ‘brain activity’ [AND] ‘psychopathology’ [OR] ‘externalizing problems’ [OR] ‘internalizing problems’ [AND] ‘Executive function’ [OR] ‘Executive functions’ [OR] ‘Executive functioning’ [OR] ‘EF’. The references of eligible studies and relevant reviews were also searched using a snowballing technique. The search yielded the following results across the databases: Web of Science, 524 articles; Scopus, 14; PubMed, 9; and Google Scholar, 1298.

During the selection process, two independent reviewers (N.B.I. and A.A.M.) performed quality assessments, identifying and removing duplicate articles due to repeated entries across different databases or languages. This resulted in a total of 66 articles, of which only 10 met the established inclusion criteria. To determine the suitability of these 10 articles for the review, their quality was assessed using the Newcastle–Ottawa Scale (Wells *et al*., 2000) and the adapted version for cross-sectional studies by Herzog *et al*. (2013) (Supplementary Materials Table S2). This scale provides a scoring system to compare studies based on factors such as study design, analysis, and presentation. To avoid the risk of biases, any disagreements between reviewers were discussed with J.M.M-M., resulting in a consensus on the selection of studies. The quality assessment process was overseen by C.B.-T. Once the quality check was completed, an exhaustive review of the selected articles was conducted to make a final selection decision.

**Figure 1.**
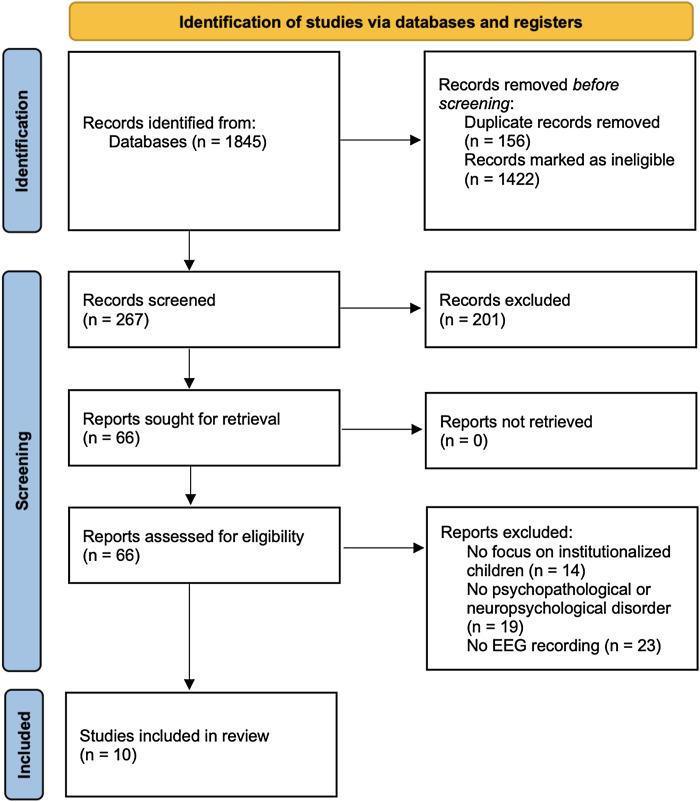
PRISMA flow diagram of systematic search and study selection according to Page *et al*. (2021).

This complex process of identification, search, and inclusion is clearly set out in the diagram in Fig. 1.

### Data extraction and analysis

A piloted data extraction form was used to extract data from eligible articles, which were reviewed simultaneously and independently by two reviewers (J.M.M.M and N.B.I). Various data points were extracted from each of the articles: authors’ names, date of publication, the country where the research took place, objective of the study, number of participants including average age and gender of the sample, access to the sample, and the situation of the minors, as well as the instruments for evaluating and measuring brain activity, the conditions under which the measurements were made, and the results for each article.

## Results

Following the inclusion criteria and after an exhaustive review, we only included and reviewed 10 articles.

### Description of the results concerning the main data of the type of study of the articles reviewed

Regarding the objectives of the research work, the results show that all the articles investigate the relationship between psychopathological and/or neuropsychological variables in institutionalised minors. However, the study typology varies according to the nature of the sample. There are two clearly differentiated groups. First, those studies that conducted follow-ups of institutionalised minors through longitudinal designs, such as (1) Troller-Renfree *et al*. (2016), Wade *et al*. (2019), Buzzell *et al*. (2020), Debnath *et al*. (2023), Tan *et al*. (2023), and Wade *et al*. (2023). The samples in these studies were initially selected and evaluated in the Bucharest Early Intervention Project (BEIP) (see Zeanah *et al*., 2003) but followed completely independent objectives and subsequent evaluations. These studies included a control group of never-institutionalised minors (NIG) and consistently involved random assignment, which strengthened internal validity by assigning participants either to continued institutional care or foster care intervention groups (CAUG and FCG, respectively). Additionally, (2) the longitudinal study carried out by Bick *et al*. (2022) focused on a sample of internationally adopted children, without a non-adopted control group, thus limiting comparisons to within-group analyses. On the other hand, we have cross-sectional studies, which include (1) independent research conducted without a connection to any other study (An *et al*., 2020), comparing adults with histories of institutionalisation (IC) to those raised in biological families (BFC) as a control group, without random assignment; (2) a cross-sectional study using a video game simultaneously with EEG recording (Hevia-Orozco *et al*., 2017), with EEG data later compared with standardised questionnaires (Hevia-Orozco and Sanz-Martin, 2018), using a control group of non-institutionalised adolescents, without random assignment; and (3) a cross-sectional study measuring mediofrontal theta power (MFTP) during a ‘go/no-go’ task with a sample from the BEIP study (Wade *et al*., 2023), including a randomised control group of NIG. The studies demonstrate good methodological rigor, supporting the reliability of their findings on the relationships between psychopathological and neuropsychological variables in institutionalised minors.

In terms of effect sizes, all studies report not only statistical significance but also various effect size measures, such as η^2^, Cohen’s d, and standardised coefficients (β), to indicate the magnitude of observed effects. For example, studies like An *et al*. (2020) and Debnath *et al*. (2023) provide effect sizes to quantify differences in neuropsychological outcomes, while others, such as Troller-Renfree *et al*. (2016); Buzzell *et al*. (2020); and Tan *et al*. (2023), use path analysis or regression models to capture the strength of associations between neuropsychological markers and psychopathological outcomes. This consistent reporting of effect sizes strengthens the interpretation of findings by highlighting the impact of institutionalisation on cognitive and emotional development.

### Description of the results with respect to the instruments and recorded brain region

All the articles used instruments for neuropsychological and/or psychopathological evaluations, with the most common being the Flanker test (Troller-Renfree *et al*., 2016; Debnath *et al*., 2023), the Health and Behaviour Questionnaire (HBQ) (Troller-Renfree *et al*., 2016; Buzzell *et al*., 2020; Debnath *et al*., 2023), and those used by Hevia-Orozco *et al*. (2017) and Hevia-Orozco and Sanz-Martin (2018).

Focusing on the analysed variables, we can see that there is variability in the characteristics, symptoms, or studied domains in each research study. A percentage of the studies focused on the cognitive and neuropsychological functions of institutionalised minors. The study carried out by Tan *et al*. (2023) focused on the mental subscale of the Bayley Scales of Infant Development (BSID-II), which measures cognitive skills including perceptual acuity, discriminations, memory, learning, communication, and abstract thinking skills, as well as intelligence quotient (IQ), assessing cognitive skills in four domains: perceptual reasoning, verbal comprehension, working memory, and processing speed in a situation of psychosocial deprivation experienced in institutions. An *et al*. (2020) evaluated the non-verbal intelligence and inhibitory control in aspects of early psychosocial deprivation produced in institutions. Wade *et al*., (2019, 2023) evaluated attention and short-term visual memory, spatial planning and problem solving, working memory and visual-spatial memory, and new learning in cases of early neglect in institutions; additionally, in Wade *et al*. (2023), they also analysed stressful life events.

Other studies focused on symptoms or psychopathological disorders. Troller-Renfree *et al*. (2016) and Debnath *et al*. (2023) analysed internalising (depression, separation anxiety, and overanxious behaviour) and externalising (oppositional defiance, conduct problems, overt aggression, relational aggression, inattention, and impulsivity) behaviour, as well as the inhibition of responses on an executive functions level in environments of early psychosocial deprivation produced in institutions. Buzzell *et al*. (2020) assessed general psychopathology as did the previous studies, but employing a latent bifactor model in which a single factor captured the shared variance between domains of psychopathology (i.e. the ‘P factor’). Bick *et al*. (2022) also focused on children’s behavioural and emotional problems, in particular evaluating social competences, problems of internalising behaviour (anxiety, depression, somatic complaints and isolation), externalising behaviour (aggressive behaviour and criminal conduct) and a mixed factor (social problems, of both thought and attention) in early adverse environments produced in institutions. Finally, in the works of Hevia-Orozco *et al*. (2017) and Hevia-Orozco and Sanz-Martin (2018), they evaluated the internalising symptoms (anxiety, depression, and post-traumatic stress disorder) in institutionalised minors.

Another of the objectives of the article was to observe the relationship between the psychopathological and neuropsychological symptoms with the oscillations in the brain activity measured by an EEG. All the studies registered the general basal spectrum of the principal brain waves (delta, theta, alpha, beta, and gamma) (Tan *et al*., 2023). However, for the analyses, some of them focused exclusively on theta (Debnath *et al*., 2023), alpha (Wade *et al*., 2019), carried out event-related potentials (ERPs) (Troller-Renfree *et al*., 2016; An *et al*., 2020), or registered MFTP while carrying out a go/no-go task (Buzzell *et al*., 2020; Wade *et al*., 2023). An increase in the slow waves (i.e. theta) was obtained as a result (Buzzell *et al*., 2020; Bick *et al*., 2022; Debnath *et al*., 2023; Tan *et al*., 2023; Wade *et al*., 2023), or a more immature EEG spectral power (Bick *et al*., 2022) in the institutionalised minors who had experienced early psychosocial deprivation. These minors presented lower results in such domains as IQ (Tan *et al*., 2023), attention (Wade *et al*., 2019; An *et al*., 2020), visual memory, spatial planning, problem solving (Wade *et al*., 2019, 2023), and inhibition (Troller-Renfree *et al*., 2016; Debnath *et al*., 2023), as well as a greater risk of experiencing externalising and internalising problems (Troller-Renfree *et al*., 2016; Hevia-Orozco and Sanz-Martin, 2018; Bick *et al*., 2022). Regarding decision-making, not all studies found difficulties in institutionalised minors (Hevia-Orozco *et al*., 2017).

Below is a description of the main data and results of each article (Tables 1 and 2).

**Table 1.** Objectives of the research works, population, and evaluation instruments

Author & year	Country	Objective of the article	Sample (SD)	Sex (%)	Age M (SD)	Study quality
Troller-Renfree *et al*. (2016)	USA	To investigate the impact of psychosocial deprivation on behavioural and neural responses (event-related potentials: *ERPs*) to assessing error monitoring and the relations between these measures and psychopathology for 12-year-old children	−136 children -A: 50 care as usual group (CAUG). -B: 50 foster care group (FCG). -C: 48 never-institutionalised group (NIG)	Female -A: 46 -B: 48 -C: 54.17	-A: 11.39 -B: 11.92 -C: 12.14	Good
Hevia-Orozco *et al*. (2017)	Mexico	-To examine the EEG correlation during a social decision-making task between a group of institutionalised adolescents and a group with no history of institutionalisation	A: 10 institutionalised adolescents (INST) B: 10 never-institutionalised group (NINST)	A: Male	A:14.8 (1.033) B:14.1 (0.783)	Good
Hevia-Orozco and Sanz-Martin. (2018)	Mexico	-To correlate the cortical electroencephalographic (EEG) and psychopathological parameters	A: 10 institutionalised adolescents (IA) B:10 never-institutionalised adolescents (NIA)	A: Male	A:14.8 (1.033) B:14.1 (0.783)	Good
Wade *et al*. (2019)	USA	-To evaluate the longitudinal trajectories of memory and executive functions from childhood in institutionalised children. -To examine whether resting EEG *alpha* power in middle childhood is associated with better memory and EF outcomes across this formative period of development	−161 children -A: 47 care as usual group (CAUG). -B: 52 foster care group (FCG). -C: 62 never-institutionalised group (NIG)	Female -A: 46.8% -B: 53.8% -C: 48.8%	Age entered institution, -A: 1.95 -B: 2.72 -C: NR	Good
An *et al*. (2020)	Russia	Investigated the long-term effects of institutionalisation on the inhibitory control of young adults raised in orphanages using the colour-word Stroop task	A: 28 biological family care (BFC) group) B: 24 institutional care (IC) group	NR	A: 22.25 (4.9) B: 22.17 (6.7)	Good
Buzzell *et al*. (2020)	USA	To explore the relationship between error-related midfrontal *theta* and psychopathology in institutionalised minors with follow-up at ages 12 and 16	−136 children - A: Care as usual group (CAUG). - Foster care group (FCG). -Never-institutionalised group (NIG)	NR	A: 16.72 (0.44) B:16.64 (0.61) C: 17.06 (0.65)	Good
Bick *et al*. (2022)	USA	Study the potential of EEG in institutionalised minors and its relation to behavioural problems	35 children	21 (60%, female)	A: Age 10 and 46 months; 23.01 (9.92) B: 8.71 (0.31)	Good
Debnath *et al*. (2023)	Germany	Examine the continuous effect of foster care intervention on the development of error monitoring in adolescents removed from institutions and placed into foster care in infancy and the emergence of psychopathology in adolescence	136 children - Care as usual group (CAUG). - Foster care group (FCG). -Never-institutionalised group (NIG)	At 16 years, 47 CAUG (22 female), 49 FCG (23 female), and 48 NIG (27 female)	A: 6–31 months (BEIP) B: 16 years of age on average	Good
Tan *et al*. (2023)	USA	Evaluate the efficacy of foster care as an alternative to residential care	136 children -Care as usual group (CAUG). -Foster care group (FCG). -Never-institutionalised group (NIG)	51.49% female	A: 6–31 months (BEIP) B: 18 years of age on average	Good
Wade *et al*. (2023)	Canada	To examine whether family care following early-life deprivation buffered the association between stressful life events and executive functioning in adolescence	−143 children -Care as usual group (CAUG). -Foster care group (FCG). -Never-institutionalised group (NIG)	At 16 years, 47.8 CAUG (24 female), 25 FCG (52.1 female), and 61.2 NIG (30 female)	16 years of age on average	Good

*Note:* The ‘Study Quality’ category was assessed using the Newcastle–Ottawa Scale. BEIP, Bucharest Early Intervention Project; EF, executive functions.

**Table 2.** Variables, instruments, and results of the articles

Author and year	Instruments used to measure psychological/neuropsychological results	Instruments used to measure neurophysiological results	Brain region/waves	Moment of measurement	Main findings	Follow-up
Troller-Renfree *et al*. (2016)	-Flanker task (using E-Prime 2.0 software) -The Health and Behaviour Questionnaire (HBQ)	-Continuous EEG was recorded using a 64-channel Geodesic Sensor Net. -EEG filters set at 0.3 and 30 Hz -Impedances were kept below 50 kΩ.	-Event-related potentials (*ERPs*)	-The basal EEG was collected at the start of the BEIP study. -The Flanker Task Test and The Health and Behaviour Questionnaire (HBQ) were evaluated at the end of the study after the minors reached the average age of 12 years.	−12-year-old children who had experienced institutionalised care showed decreased accuracy on a modified Flanker task when compared to their never-institutionalised peers. -Perturbed error monitoring may represent a risk for externalising problems in previously institutionalised children.	Yes
Hevia-Orozco *et al*. (2017)	-The brief form of the Wechsler Intelligent Scales* -NEUROPSI A-M* [*as inclusion criterion] -The Children’s Depression Inventory (CDI) -The Children’s Post-Traumatic Stress Scale-Spanish version (CPSS) -The Spence Children’s Anxiety Scale-Spanish version (SCAS)	- The EEG signals were digitally amplified using a Neuroscan-NuAmps amplifier, with filter settings ranging from 1 to 50 Hz. -EEGs were analysed by the EEG Bands computer program. -Impedances were kept below 10 kΩ.	-Fp1–Fp2, F3–F4,T3,T4, F3–P3, F4–P4. -*Delta, Theta, Alpha, Beta1, Beta2, Gamma*	The brief form of the Wechsler Intelligent Scales and NEUROPSI A-M as screening and the rest of the questionnaires at the time of recording the EEG. -The Ultimatum Game was used simultaneously with the recording of the EEG.	-There were no differences between both groups in behavioural parameters (decision-making). -The INST group exhibited a stronger correlation in *delta* and *alpha-1* frequencies during the acceptance and rejection of proposals, respectively, between the Fp1 and F3 regions. -This group also demonstrated a reduced *gamma* correlation between the F3 and T3 regions during both the acceptance and rejection of proposals. -During the acceptance of proposals, the INST group showed a lower *gamma* correlation between the F3 and P3 regions and a higher *delta* correlation between the F4 and P4 regions.	No
Hevia-Orozco and Sanz-Martin. (2018)	-The brief form of the Wechsler Intelligent Scales -NEUROPSI A-M -The Children’s Depression Inventory (CDI) -The Children’s Post-Traumatic Stress Scale-Spanish version (CPSS) -The Spence Children’s Anxiety Scale-Spanish version (SCAS)	-In the EEG analyses, a *t*-test for the RP values of each electrode recorded in each frequency band was carried out. No additional information is given.	-Fp1–Fp2, F3–F4, Fp1–F3, Fp2–F4, F3–T3, F4–T4, F3–P3, F4–P4. -*Delta, Theta, Alpha, Beta1, Beta2, Gamma*	-The brief form of the Wechsler Intelligent Scales and NEUROPSI A-M as screening and the rest of the questionnaires at the time of recording the EEG.	-The IA group showed higher indices of depression, anxiety and PTSD, accompanied by low relative power (RP) in the fast bands, high activity in the slow bands in frontal areas, and higher *alpha2* RP in temporal areas. -EEG activity in different areas in adolescents might reflect abnormal development or lack of development, such us the increased *theta* RP observed in IA.	No
Wade *et al*. (2019)	-Cambridge Neuropsychological Test Automated Battery (CANTAB)	-The EEG recording system from James Long Company - The EEG signal was amplified with a gain of 5,000 and filtered using a bandpass range of 0.1–100 Hz. -Electrode impedances were kept at <10 kΩ.	-F3, F4, Fz, C3, C4, P3, P4, Pz, O1, O2, T7, T8 -*Alpha*	-The basal EEG was collected at the start of the BEIP study. In the initial evaluation, the children were between 6 and 31 months. -The neuropsychological measurements were evaluated at 8 years of age, at 12 and at 16.	- Early-emerging disparities in attention and short-term visual memory, as well as spatial planning and problem solving, between ever- and never-institutionalised children persisted through adolescence - The gap in spatial working memory between ever- and never-institutionalised children widened by adolescence -Early difficulties in visual-spatial memory and new learning among children in foster care were mitigated by adolescence. -The secondary analyses showed that a greater power of *alpha* in the EEG in repose at 8 years of age predicted better results of the FE in several domains at the age of 8, 12 and 16 years.	At age 8, 12, and 16
An *et al*. (2020)	-The Culture-Fair Intelligence Test, CFIT - Stroop Test (There were four emotional Stroop conditions and two classic Stroop conditions in the current experiment)	-The EEG recording system from the actiCHamp amplifier (BrainProducts, Inc.) -The data were preprocessed offline using Brain Vision Analyser soft- ware v 2.1 (BrainProducts Inc.). -EEG data were down-sampled to 250 Hz and bandpass filtered (0.1–30 Hz) and impedances were kept below 25 kΩ.	-AF3, AFz, AF4, F1, Fz, F2, FC1, C1, CP1, FCz, Cz, CPz, Fc2, C2, CP2, P1, PO3, POz, PO4, P2, Pz. -Event-related potentials (*ERPs*)	-Only a single time of measurement.	-The analysis of the reaction time showed that the institutional care group (IC) was in general slower than the BFC in the Stroop test, but the absence of group differences in the condition of interference may be explained by the low complexity of the Stroop test in the study in question. -The IC group showed a reduced *P3* event-related potential component on both the congruent and incongruent trials. Findings suggest general attention difficulties in this population, rather than inhibitory control deficits.	No
Buzzell *et al*. (2020)	-MacArthur Health and Behaviour Questionnaire (HBQ) -Go/no-go task	-EEG data were collected via a 64-channel HydroCel Geodesic Sensor Net and EGI software (Electrical Geodesic, Inc., Eugene, OR). -All EEG analyses were performed using the EEGLAB toolbox -Electrode impedances were lowered to<50 kΩ -Data were sampled at 250 Hz (filtered at 0.3 Hz- 49)	-Mediofrontal *theta* power (*MFTP*)	-At 12 years of age and at 16 years of age on average in the sample.	-Children with prolonged psychosocial deprivation exhibit impaired task-related mediofrontal *theta* -Earlier placement into foster care was associated with higher levels of mediofrontal *theta*, as well as relatively greater behaviour following errors (PEA). -Reduced mediofrontal *theta* reflected an indirect neural pathway linking early neglect to general psychopathology. -Whereas mediofrontal *theta* was found to decrease from ages 12–16 on average, placement into foster care yielded less of a decline in mediofrontal *theta* over this period	At age 12, and 16
Bick *et al*. (2022)	-The Child Behaviour Checklist (CBCL/6-18)	-The EEG recording system from the Advanced Neuro Technology Acquisition hardware -32 channel Ag/AgCl electrodes. -EEG data were processed using the Boston EEG Automated Process- ing Pipeline and Harvard Automated Processing Pipeline for EEG and EEG data -EEG data were down-sampled to 250 Hz.	-F4, F5, Fz, F7, F8, C3, Cz, C4, P3, P4, Pz, P7, P8, Oz, Oz, O2, T7, T8. -*Theta* (4–6 Hz); *Alpha* (7–12 Hz); *Beta* (13–20 Hz); *Gamma* (21–50 Hz).	-Adoptive data at the start of the study. -Parents completed the CBCL at the 8-year follow-up visit. -Age at EEG recording was 8.71 years (SD = .31)	-A more advanced adoption age was associated with more immature or atypical profiles of the cortical function of the average child, based on a relatively higher power of *theta*, a relatively lower power of *alpha*, and a lower absolute power of *beta* and *gamma*. -A more immature spectral power of the EEG was indirectly linked to a more advanced adoption age with a greater risk of externalising problems in childhood.	7 years of age on average
Debnath *et al*. (2023)	-The Flanker Task Test -The Health and Behaviour Questionnaire (HBQ)	-The EEG Analysis system from the Electrical Geodesics Company -EEGLAB toolbox -64 channel HydroCel Geodesic Sensor Net and a NetAmps 300 amplifier -EEG data were down-sampled to 250 Hz and bandpass filtered (0.3–40 Hz) and impedances were kept below 50 kΩ.	-*Theta* (4–8 Hz) power at FCz electrodes -*ERN* (error-related negativity)	-The basal EEG was obtained at the start of the BEIP study. -The Flanker Task Test, and The Health and Behaviour Questionnaire (HBQ) were done at the end of the study after the population reached the age of 16 years. Other previous evaluations at 8 and 12 years of age (McDermott *et al*., 2013; Troller-Renfree *et al*., 2016).	−16-year-old adolescents who as young children were randomised to foster care (FCG) exhibited enhanced response accuracy and processing speed on a modified Flanker task when compared to those who experienced care as usual (CAUG). -Children who experienced prolonged psychosocial deprivation exhibit impaired *ERN* in adolescence. -The findings suggest that deficits in error monitoring are associated with externalising behaviour problems, and continued high-quality foster care might mitigate the externalising problems in previously institutionalised children	16 years of age on average
Tan *et al*. (2023)	-The Bayley Scales of Infant Development (BSID-II) [study focused on the mental subscale] -The Weschler Intelligence Scale for Children, fourth edition (WISC-IV)	-The EEG Analysis System from the James Long Company -EEG data were processed using the EEGLAB toolbox - EEG data were down-sampled to 250 Hz and bandpass filtered (0.3–50 Hz). - Impedances of all sensors were kept below 10 kΩ.	-F3, F4, Fz, C3, C4, P3, P4, Pz, O1, O2 -For relative *theta, alpha, beta,* and *gamma* power across baseline	-The basal EEG was taken at the start of the BEIP study; baseline (M = 20.40 months; SD = 7.20), 30 months (M = 30.84 (2.04), and 42 months (M = 42.36 (1.44). -The BSID-II was administered at the start of the study. - The WISC-IV at the end of the study after reaching the age of 18 years.	-Growing up in institutions predicted a greater power of *theta* in repose at the start of the study, which in turn predicted a lower IQ at 30 and 42 months, and later a lower IQ at 18 years of age (this increases in late placings). -No effects of mediation were found for the *alpha*, *beta* and *gamma* bands. - The analysis of the WISC-IV replicated the effects found with the CI on a large scale within the domains of perceptive reasoning, working memory and processing speed.	16 years of age on average
Wade *et al*. (2023)	-Stressful life events (SLEs) -The CANTAB -Go/no-go task	-The EEG Analysis system from the Electrical Geodesics Company -64 channel HydroCel Geodesic Sensor Net	-Mediofrontal *theta* power (*MFTP*) -Cluster of mediofrontal electrodes (Electrode E4/FCz and the two nearest electrodes)	-The data were gathered at the time of the study.	-Findings provide preliminary evidence that family care following early deprivation may facilitate resilience against stress during adolescence on EF. -We show that greater exposure to SLEs at 16 years is associated with reduced EF and *MFTP* among youth who experienced prolonged institutional care, but not those who were randomly assigned to foster care intervention early in childhood or never-institutionalised youth -More independent SLEs predicted lower EF and more dependent SLEs predicted lower *MFTP*, but only among adolescents with prolonged early deprivation.	16 years of age on average

*Note:* NR, no response; DQ (Development Quotient[age equivalent score /- chronological age] × 100); *ERN* (error monitoring underlies a well-established *ERP* component known as error-related negativity (ERN), which is a negative deflection that is maximal at the frontocentral scalp sites and peaks within the first 100 ms following an error response); error monitoring works in tandem with inhibitory control by signalling and detecting errors in order to optimise behaviour goals (McDermott *et al*., 2013; Moser *et al*., 2013); EF, executive functions.

## Discussion

The review provides a detailed description of the use of electroencephalographic techniques and psychological and/or neuropsychological tests as tools to gain greater knowledge of the functioning of the brains of institutionalised minors and to assist in the detection of the psychological and neuropsychological consequences present in these children. One of the main conclusions drawn from the review is that the psychological health of institutionalised minors is significantly impacted. As Gunnar *et al*. (2001) point out, the heterogeneous and often co-existing aspects of deprivation can be understood on three levels: first, the lack of attention to basic needs, such as adequate health and nutrition; second, the lack of appropriate stimulation necessary for sensorimotor, cognitive, linguistic, and social development; and finally, the lack of stability and consistent relationships with significant adults, with whom to form emotional attachment. All these circumstances are reflected in the reviewed articles, in which the minors come from foster care (Troller-Renfree *et al*., 2016; Wade *et al*., 2019; An *et al*., 2020; Buzzell *et al*., 2020; Bick *et al*., 2022; Debnath *et al*., 2023; Tan *et al*., 2023; Wade *et al*., 2023) and even, in some cases, having been victims of abuse (Hevia-Orozco *et al*., 2017; Hevia-Orozco and Sanz-Martin, 2018). The background of physical, psychological, or sexual abuse suffered by these minors was probably greater than what is reflected in these studies, as pointed out by McDonald and Brook (2009), who stated that 55% of those entering care centres had suffered child abuse or neglect.

One of the most relevant studies is the BEIP, begun in 2001 by Zeanah *et al*. (2003). From this study arises part of the main sample used in the initial evaluations of several longitudinal studies included in this review (Troller-Renfree *et al*., 2016; Wade *et al*., 2019; Buzzell *et al*., 2020; Debnath *et al*., 2023; Tan *et al*., 2023; Wade *et al*., 2023). Although these studies share participants from the same original sample, each study pursued distinct objectives and conducted independent evaluations over the years. This study analysed the efficacy of foster care as an alternative to residential care. To do so, researchers included minors randomly assigned to foster homes, minors in residential care, and minors who were never institutionalised. The study lasted 54 months. However, as pointed out in our review, the minors were followed in a longitudinal manner, and the current research shows data from the evaluations of these groups at 12 and 16 years of age. During these years, many of the minors left institutional care for varying reasons. Nevertheless, throughout these decades, measurements were still being taken of behavioural, neuropsychological and psychopathological symptoms, as well as EEG. From this study (Zeanah *et al*., 2003), many later studies have arisen oriented towards studying a great number of variables, such as intelligence (Almas *et al*., 2016), psychopathology (Humphreys *et al*., 2015), cognitive skills (Humphreys *et al*., 2022), the neurobiology of the processing of emotions (Moulson *et al*., 2009), alterations in the structure of the brain (Vanderwert *et al*., 2010), or electroencephalographic comparisons with other minors (Marshall *et al*., 2004). All of them include psychosocial deprivation occurring in institutions as a transversal theme; yet, in the review, we have focused solely on those studies with psychological and neuropsychological variables, as well as EEG measurements.

As for these variables, the minors who presented normative brain development were characterised by a decrease in the power of the lowest frequency EEG and an increase in the power of the highest frequency bands (Marshall *et al*., 2002; Anderson and Perone, 2018). Comparing these data with the results of the samples from our review shows that institutionalised minors have a general immaturity of the EEG, with slow waves predominating, principally in the theta band (Bick *et al*., 2022; Debnath *et al*., 2023; Tan *et al*., 2023), consistent with research into babies and children who have received institutional care (Vanderwert *et al*., 2010). This pattern is seen even in specific areas, such as the frontomedian (Buzzell *et al*., 2020; Wade *et al*., 2023), which is involved in inhibition, detection, and conflict resolution processes, as well as performance monitoring (Badgaiyan and Posner, 1997; Fu *et al*., 2022). Furthermore, the results obtained with the ERN in institutionalised minors are consistent with studies showing that early exposure to prolonged adversity and neglect can lead to a lasting decrease in neuronal error processing (McDermott *et al*., 2013; Frenkel *et al*., 2020), even exhibiting a reduction in the ERN after possible adoption or fostering (Loman *et al*., 2013), or following child sexual abuse and/or neglect (Letkiewicz *et al*., 2023). These findings may indicate continuous deficits in the development of brain circuits involved in error monitoring. Nevertheless, it cannot be concluded with any certainty that the early changes in brain activity can predict worse long-term cognitive results, despite the existence of data demonstrating that the changes in the EEG are related to certain cognitive dysfunctions (Troller-Renfree *et al*., 2016; Hevia-Orozco *et al*., 2017; Hevia-Orozco and Sanz-Martin, 2018; An *et al*., 2020; Buzzell *et al*., 2020; Bick *et al*., 2022; Debnath *et al*., 2023; Tan *et al*., 2023). In order to determine how early brain activity is related to long-term cognitive development, it is important to examine the interaction between the cognitive and brain activity on multiple occasions, as done in the longitudinal studies carried out by the BEIP (Zeanah *et al*., 2003) and by Yarger *et al*. (2020), or to start new studies that include recent advances in the EEG, or to incorporate other neuroimaging techniques with spatial resolution in the initial stages of development (Hodel *et al*., 2015).

As for the consequences or the impact of institutionalisation on the executive functions (Johnson *et al*., 2021), in the reviewed articles, the minors present worse results in working memory and processing speed (Debnath *et al*., 2023; Tan *et al*., 2023), as well as in inhibition (An *et al*., 2020). However, no significant problems were found in decision-making (Hevia-Orozco *et al*., 2017). One of the most relevant questions arising from this review is whether the executive functions could mediate between early psychosocial deprivation and psychopathology in institutionalised minors (Wade *et al*., 2019; Buzzell *et al*., 2020). A possible explanation is that the frontoparietal network, which sustains the executive functions, is altered in many psychopathological disorders (Sha *et al*., 2019), and children who have experienced early psychosocial deprivation show an altered function and structure in this very region (McLaughlin *et al*., 2019). It can thus be concluded that inadequate environments over critical periods can fundamentally change the dynamics of the neurocognitive development (Nelson and Gabard-Durnam, 2020). Some research works have also demonstrated that institutionalised minors have a lower IQ in comparison to those minors who were in foster care, from early infancy to the age of 18 years (An *et al*., 2020; Tan *et al*., 2023). Removing a minor from institutional care may allow for recovery of IQ to normal ranges if they receive adequate stimulation (Nelson *et al*., 2007; Sonuga-Barke *et al*., 2017). However, there are factors that could intervene in the said improvement in IQ, such as fostering at an early age and the quality of care the minor receives in the foster family (Nelson *et al*., 2007; Bos *et al*., 2009; Almas *et al*., 2016; Wade *et al*., 2019; Humphreys *et al*., 2022).

Psychopathological symptoms can also be sensitive to institutionalisation. Institutionalised minors have a greater risk of suffering from psychopathological disorders and symptoms (McLaughlin *et al*., 2010; Hevia-Orozco and Sanz-Martin, 2018). The electroencephalographic power, through the relative and absolute power in the alpha, beta, and gamma bands indirectly explains the links between a longer time being institutionalised and a greater risk of externalising problems (Hevia-Orozco *et al*., 2017; Hevia-Orozco and Sanz-Martin, 2018; Wade *et al*., 2019), but not of internalising in infancy (McLaughlin *et al*., 2010; Bick *et al*., 2022). This would suggest that early adversity in institutional care could intervene specifically in the neuronal systems involved in externalising problems related to disruptive behaviour, attention, and impulsiveness. These effects are consistent with other studies involving institutionalised children with the risk of externalising problems and the presence of attention deficit and hyperactivity disorders (McLaughlin *et al*., 2010), with a deficient interpersonal functioning (Almas *et al*., 2012) as well as executive dysfunctions and bad behaviour regulation (Tarullo *et al*., 2011). Another finding linking psychopathology with EEG variations is that a reduction in MFTP reflects an indirect neuronal path connecting early institutional neglect with general psychopathology between 12 and 16 years of age and not solely with internalising or externalising problems. MFTP reduction is less pronounced in fostered minors than in those institutionalised. At the same time, the changes in the development of the theta mediofrontal predict greater reductions in the general psychopathology when care involves fostering (Buzzell *et al*., 2020; Wade *et al*., 2023).

In this review, we have verified that the duration of institutionalisation can be a key factor in cognitive development (Rutter and O’Connor, 2004; Wade *et al*., Buzzell *et al*., 2019; 2020). Prolonged institutionalisation is associated with reduced theta power, indicating general immaturity in brain development (Debnath *et al*., 2023). Moreover, other studies have found that children in residential care who were subsequently fostered can achieve functional levels similar to normotypical children, overcoming delays in cognitive and neurocognitive development (Colombo *et al*., 1992; O’Connor *et al*., 2003). Compared to the review by Perego *et al*. (2016), our review represents a 9-year update of scientific advances, incorporating more recent studies and more precise methodologies. First, we delve into the data collection methods, describing in detail the questionnaires used for psychological and neuropsychological assessment. Second, we focus on the techniques employed in EEG spectral analysis, including evoked potentials, MFTP, error-related negativity (ERN), and baseline activity. In addition, we provide a detailed description of the brain regions assessed and the types of waves recorded, providing a more specific analysis of the neurophysiological findings. Finally, unlike the previous review, our selection criteria clearly establish the need for included studies to jointly address psychopathology, neuropsychology, and EEG, ensuring a comprehensive approach to assessing the impact of institutionalisation on child development. Furthermore, the reviewed evidence indicates that early detection of EEG abnormalities in institutionalised children could help identify those at greater risk of developing neuropsychological and psychopathological difficulties, facilitating targeted interventions aimed at improving emotional regulation and executive functions (Marshall *et al*., 2004; Vanderwert *et al*., 2010, 2016; Law *et al*., 2023). Moreover, creating more stimulating environments and reducing the duration of institutionalisation could mitigate the negative effects observed in EEG, reinforcing the importance of foster care models and individualised care in child development (Vanderwert *et al*., 2016; Wade *et al*., 2019; Debnath *et al*., 2023).

The information on the subject being dealt with in this review is novel but insufficient, as there are still only very few empirical studies that deal with such a complex subject. There are, therefore, more reasons to continue investigating in the future. It would also be a good idea to analyse the utility of other questionnaires, to attempt to replicate previous studies in order to be able to verify the results with larger samples, as pointed out by other authors (Tan *et al*., 2023). Future research should prioritise longitudinal studies that analyse the evolution of EEG activity alterations and their impact on long-term neuropsychological symptoms. Specifically, it would be relevant to examine whether specific patterns, such as MFTP, impact the development of executive functions and whether impaired error monitoring remains a predictor of externalising problems in previously institutionalised adolescents (Troller-Renfree *et al*., 2016). To overcome these limitations, it would be advisable to conduct repeated EEG measurements with cognitive control tasks, such as the Flanker Task, administered at different developmental stages (e.g. at ages 12 and 16) to assess changes in inhibition and cognitive control. Additionally, combining EEG with functional neuroimaging techniques would allow for a more precise analysis of the effects of institutionalisation on brain structure and function. Finally, expanding sample sizes could help improve our understanding of how institutionalisation affects brain development, cognitive performance, and mental health.

Finally, we should mention one of the possible limitations of our study: that doctoral and masters theses were not included (Oliveira, 2016), as were other studies that, although they did not comply with the established inclusion criteria, could have been relevant, such as, for instance, those that did not take measurements of the basal activity of the EEG (Humphreys *et al*., 2022) or that only used measurements of the EEG (Vanderwert *et al*., 2016; Debnath *et al*., 2020; Sheridan *et al*., 2022). Moreover, many of the studies included in this review are from specific samples, such as the BEIP, which may limit the generalisability of the results to other cultural and institutional contexts. Future research should be expanded to diverse populations to assess the broader applicability of these results. We could also have included some articles that studied other domains outside the objective of our study but that could also have been relevant, as is the case of the research carried out by Chinn *et al*. (2021), who evaluated the development of the processing of emotions in prosody and the processing of prosody in speech, as well as Young *et al*. (2017), who focused on the processing of facial emotions.

## Conclusion

This review highlights the limited research on the impact of institutionalisation on brain activity and its relationship with psychopathological and neuropsychological symptoms. A total of 10 studies met the inclusion criteria, providing evidence that institutionalised minors exhibit altered EEG patterns, particularly an increase in slow-wave activity, which has been linked to cognitive impairments and a higher risk of externalising symptoms. The results of this study can be used to mitigate the negative effects identified in EEG activity at three different levels of prevention. First, preventing situations of child neglect (e.g. family adversity, substance use, etc.) that increase the likelihood of institutionalisation is crucial, given that prolonged institutionalisation has been associated with neurophysiological alterations, such as decreased activity in fast bands and increased activity in slow bands. Second, the early detection of neuropsychological and psychopathological deficits through systematic assessments allows for early intervention to minimise the impact of institutionalisation on the development of the nervous system (An *et al*., 2020). Finally, developing interventions adapted to life in institutions, focused on stimulating affected cognitive functions and improving emotional regulation, may further reduce these negative outcomes. The available evidence suggests that foster care models and individualised care can act as resilience factors, reducing the adverse effects of institutionalisation on child development (Bick *et al*., 2022; Wade *et al*., 2023). These findings reinforce the need for child protection policies to integrate evidence-based approaches to improve the care and development of institutionalised children.

## Supporting information

Barbosa-Torres et al. supplementary material 1 Barbosa-Torres et al. supplementary material

Barbosa-Torres et al. supplementary material 2 Barbosa-Torres et al. supplementary material

## Data Availability

No new data were created or analysed in this study.

## Supplementary material

The supplementary material for this article can be found at https://doi.org/10.1017/neu.2025.19
