# Supplementary material for: Electroencephalogram activity related to psychopathological and neuropsychological symptoms in institutionalised minors: a systematic review
Source: Acta Neuropsychiatr. 2025 May 8;37:e62. doi: 10.1017/neu.2025.19 (PMC13130352; doi:10.1017/neu.2025.19)
Supplement: Barbosa-Torres et al. supplementary material 2 — Barbosa-Torres et al. supplementary material [file S0924270825000195sup002.docx]

# Newcastle-Ottawa Scale for cohort studies

| Study | Selection | | | | Comparability | Outcome | | | Total Score | Quality |
| --- | --- | --- | --- | --- | --- | --- | --- | --- | --- | --- |
|  | Representativeness of the exposed cohort | Selection of the non exposed cohort | Ascertainment of exposure | Demonstration that outcome of interest was not present at start of study | Comparability of cohorts on the basis of the design or analysis | Assessment of outcome | Was follow-up long enough for outcomes to occur | Adequacy of follow up of cohorts |  |  |
| Troller-Renfree *et al*. (2016) | 1 | 1 | 1 | 1 | 2 | 1 | 1 | 1 | 9 | Good |
| Wade *et al*. (2019) | 1 | 1 | 1 | 1 | 2 | 1 | 1 | 1 | 9 | Good |
| Buzzell *et al*. (2020) | 1 | 1 | 1 | 1 | 2 | 1 | 1 | 1 | 9 | Good |
| Bick *et al*. (2022). | 1 | 0 | 1 | 1 | 2 | 1 | 1 | 1 | 8 | Good |
| Debnath *et al*. (2023) | 1 | 1 | 1 | 1 | 2 | 1 | 1 | 1 | 9 | Good |
| Tan *et al*. (2023) | 1 | 1 | 1 | 1 | 2 | 1 | 1 | 1 | 9 | Good |
| Wade *et al*. (2023) | 1 | 1 | 1 | 1 | 2 | 1 | 1 | 1 | 9 | Good |

Note. The studies' quality was rated as poor (0-3), moderate (4-6), or good (7-9).

**Newcastle-Ottawa Scale adapted for cross-sectional studies (Herzog *et al*., 2013)**

| Study | Selection | | | | Comparability | Outcome | | Total Score | Quality |
| --- | --- | --- | --- | --- | --- | --- | --- | --- | --- |
|  | Representativeness of the sample | Sample size | Non-respondents | Ascertainment of the exposure (risk factor) | Comparability of subjects in different outcome groups on the basis of design or analysis. Confounding factors controlled. | Assessment of outcome | Statistical test |  |  |
| Hevia-Orozco *et al*. (2017) | 1 | 0 | 0 | 1 | 2 | 2 | 1 | 7 | Good |
| Hevia-Orozco and Sanz-Martin (2018) | 1 | 0 | 0 | 1 | 2 | 2 | 1 | 7 | Good |
| An *et al*. (2020) | 1 | 1 | 0 | 1 | 2 | 2 | 1 | 8 | Good |

Note. The quality of cross-sectional studies was rated as Unsatisfactory (0-4 points), Satisfactory (5-6 points), Good (7-8 points), or Very Good (9-10 points).
